# Crystal structure of [K(18-crown-6)]^+^_2_[Pt(CN)_4_]^2−^

**DOI:** 10.1107/S2056989019015238

**Published:** 2019-11-15

**Authors:** Malte Sellin, Moritz Malischewski

**Affiliations:** a Freie Universität Berlin, Institut für Chemie und Biochemie - Anorganische Chemie, Fabeckstrasse 34-36, 14195 Berlin, Germany

**Keywords:** tetra­cyano­platinate, crown ether, platinum, potassium, crystal structure

## Abstract

In the title compound, di-μ-cyanato-1:2κ^2^ *N*:*C*;2:3κ^2^ *C*:*N*-di­cyanato-2κ^2^ *C*-bis­(1,4,7,10,13,16-hexa­oxa­cyclo­octa­deca­ne)-1κ^6^ *O*;3κ^6^ *O*-1,3-dipotassium(I)-2-platinum(II), [K_2_Pt(CN)_4_(C_12_H_24_O_6_)_2_] or [K(18-crown-6)]_2_·[Pt(CN)_4_], two *trans*-orientated cyano groups of the square-planar [Pt(CN)_4_]^2−^ dianion (Pt site symmetry ) bind to one potassium ion each, which are additionally coordinated by the six O atoms of 18-crown-6. No Pt⋯Pt inter­actions occur in the crystal, but very weak Pt⋯H contacts (2.79 Å) are observed.

## Abstract 2

K_2_Pt(CN)_4_ becomes soluble in di­chloro­methane upon addition of two equivalents of 18-crown-6. Crystals of [K(18-crown-6)]_2_ [Pt(CN)_4_] are obtained upon layering the di­chloro­methane solution with di­ethyl­ether. No Pt⋯Pt inter­actions are observed in the crystal.

## Chemical context   

Polycyano­metallates are an important class of inorganic compounds with intriguing properties. As a result of their anionic nature and high nucleophilicity, they have been widely used as metallo-ligands in coordination chemistry. Depending on the geometry of the polycyano­metallate, various topologies can be realized (Alexandrov *et al.*, 2015 ▸). While photomagnetic effects have been predominantly realized with hexa- and octa­cyano­metallates (Ohkoshi *et al.*, 2012 ▸), studies on tetra­cyano­platinates and their derivatives have focused on the high electrical conductivities of mixed-valent Krogmann’s salts K_2_[Pt(CN)_4_]Br_0.32_·2.6H_2_O (Krogmann, 1969 ▸), vapochromic sensor materials (*e.g.* Zn[Pt(CN)_4_] for ammonia (Varju *et al.*, 2019 ▸) and spin-crossover compounds such as [Fe(pyrazine)][Pt(CN)_4_]·2H_2_O (Niel *et al.*, 2001 ▸). However, alkali salts of polycyano­metallates are in generally water-soluble but suffer from insolubility in organic solvents. A general way to increase the solubility of metals salts in organic solvents is the utilization of crown ethers. For example, even potassium permanganate KMnO_4_ becomes benzene-soluble by coordination of 18-crown-6 to the potassium cation (Doheny & Ganem, 1980 ▸). During our attempts to explore the coordination chemistry of the tetra­cyano­platinate dianion [Pt(CN)_4_]^2−^ in organic solvents, we realized that commercially available K_2_[Pt(CN)_4_] is insoluble in di­chloro­methane but dissolves rapidly upon addition of 18-crown-6. The product [K(18-crown-6)]_2_ [Pt(CN)_4_], which was already isolated many years ago by a rather complicated procedure (Almeida & Pidcock, 1981 ▸), could now be obtained in crystalline form. In contrast to other tetra­cyano­platinate(II) salts with large organic cations [*e.g*. PPh_4_^+^ (see Nast & Moerler, 1969 ▸) and NBu_4_^+^ (see Mason & Gray, 1968 ▸)], which are prepared by metathesis reactions in water, this new procedure makes the access to tetra­cyano­platinate salts with solubility in organic solvents even more facile.
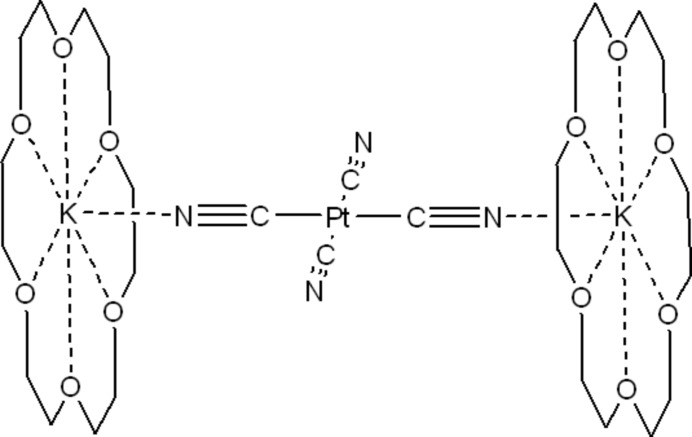


## Structural Commentary   

**Figure 1:**
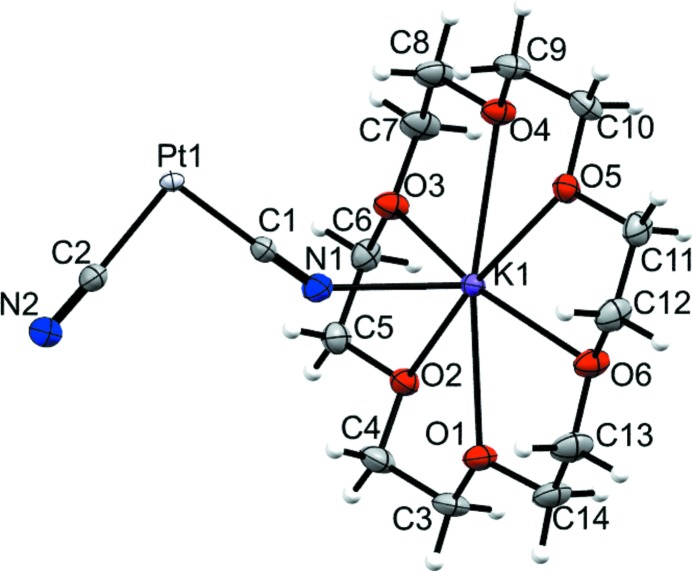
The asymmetric unit of the title compound with displacement ellipsoids shown at the 50% probability level.

[K(18-crown-6)]_2_ [Pt(CN)_4_] (Fig. 1 ▸) crystallizes in the monoclinic space group *P*2_1_/*n*. The tetra­cyano­platinate moiety displays a square-planar mol­ecular geometry with the platinum atom lying on a crystallographic inversion centre. Two *trans*-orientated cyano groups coordinate *via* their terminal nitro­gen atoms to the potassium ions in a rather bent fashion [K1—N1—C1 = 146.76 (17)°] while the Pt—C—N bonds are almost linear [Pt1—C2—N2 = 178.81 (18)°]. The Pt—C and C—N bond lengths do not differ significantly between the terminal or bridging cyano ligands [Pt1—C2 = 1.996 (2) Å *versus* Pt1—C1 = 1.991 (2) Å and C2—N2 = 1.155 (3) Å *versus* C1—N1 = 1.154 (3) Å]. The six oxygen atoms of the crown ether coordinate to the potassium ion in a hexa­gonal-planar fashion. Additionally, one apical position is occupied by a nitro­gen atom of a cyano group, although the K—N distance is relatively long [2.732 (2) Å]. The potassium ion is located 0.295 Å above the the O_6_ centroid [K—O distances = 2.769 (1)–2.837 (1) Å].

## Supra­molecular features   

**Figure 2:**
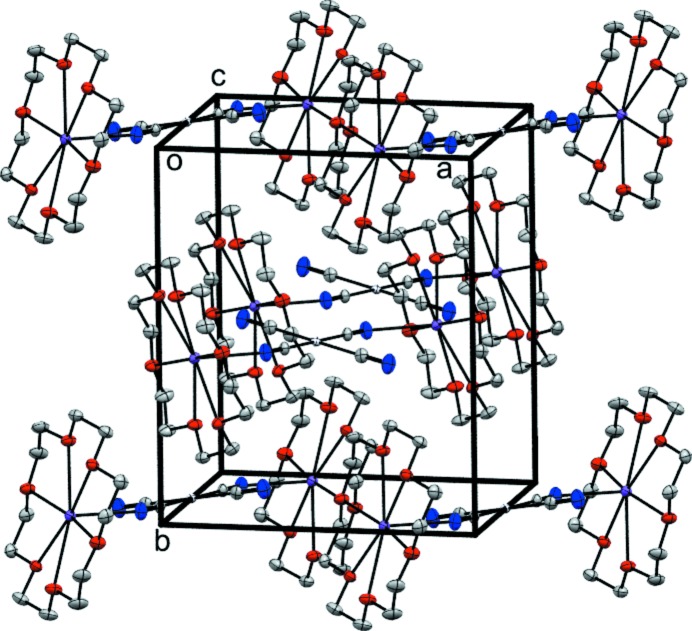
Packing in the unit cell of the title compound.

A common feature of tetra­cyano­platinate salts is the formation of columnar stacks of the planar tetra­cyano­platinate anions with Pt⋯Pt distances in the range of 3.0–3.8 Å, see, for example, Washecheck *et al.* (1976 ▸), Holzapfel *et al.* (1981 ▸), Mühle *et al.* (2004 ▸) and Neuhausen *et al.* (2011 ▸). However, in the crystal structure of the title compound (Fig. 2 ▸), no platino­philic inter­actions are observed. This is in accordance with findings of Stojanovic *et al.* (2011 ▸) who stated that large organic cations can suppress the formation of Pt⋯Pt contacts. Inter­molecular inter­actions are not very pronounced in this crystal structure. However, the two uncoordinated cyano groups each point towards one neighbouring hydrogen atom in a slightly bent fashion (C—N⋯H = 152°; Table 1 ▸) although the N⋯H distance is relatively long (2.55 Å). Moreover, two hydrogen atoms from two different crown ether mol­ecules form weak contacts to the platinum atom in a linear fashion (H⋯Pt⋯H = 180°), which results in a distorted axially elongated pseudo-octa­hedral PtC_4_H_2_ coordination environment for the platinum atom. The Pt⋯H distances are slightly smaller than the sum of the van der Waals radii (2.79 Å).

## Database survey   

A database survey (CSD version 5.40, update of November 2018; Groom *et al.*, 2016 ▸) gave 348 hits for the [Pt(CN)_4_] moiety and 1562 hits for the [K(18-crown-6)] moiety. While the tetra­cyano­platinate moiety binds to many elements from the periodic table, only a few tetra­cyano­platinate salts with metal–crown ether counter-cations are known. For example, complexes of Ba^2+^ [Pt(CN)_4_]^2−^ with 18-crown-6 (Olmstead *et al.*, (2005 ▸), dibenzo-18-crown-6 (Olmstead *et al.*, 2016)) and di­aza-18-crown-6 (Olmstead *et al.*, 2009 ▸). In the first two examples, the Ba^2+^ cation exhibits a coordination number of 10 whereas only ninefold coordination is observed in the last case. In general, these high coordination numbers result from bridging cyanide ligands and oxygen-containing donor solvents that bind to the Ba^2+^ cations. In [Tl(18-crown-6)]_2_[Pt(CN)_4_] (Liu *et al.*, 2006 ▸), only a sevenfold coordination is observed for the thallium cation. Inter­estingly, Tl^+^ does not bind to a terminal cyanide group but forms a weak metallophilic contact to Pt^2+^ (Tl⋯Pt distance = 3.185 Å).

The combination of [K(18-crown-6)] cations with other polycyano­metallates is relatively rare. Crystal structures of [K_3_(18-crown-6)_3_(H_2_O)_4_][Cr(CN)_6_]·3H_2_O (Zhou *et al.*, 2003 ▸), [K(18-crown-6)]_2_[K(18-crown-6)(H_2_O)_2_][Ru(CN)_6_]·CH_2_Cl_2_ (Vostrikova & Peresypkina, 2011 ▸) and [K(18-crown-6)]_2_[K(18-crown-6)(C_3_H_7_OH)][Os(CN)_6_]·2C_3_H_7_OH·H_2_O (Vos­tri­kova & Peresypkina, 2011 ▸) have been reported in the literature.

## Synthesis and crystallization   

Potassium tetra­cyano­platinate (37.7 mg, 0.1 mmol) was suspended in 3 ml of CH_2_Cl_2_. Then, 52.8 mg (0.2 mmol) of 18-crown-6 were added and the mixture was stirred for several minutes until the solid had completely dissolved. A small part of the solution was placed in a narrow glass tube and layered with diethyl ether. Colourless blocks of the title compound formed overnight. IR(ATR) (cm^−1^): 2898–2815 [*m*, *v*(CH)], 2126 [*s*, *n*(CN)], 1451 [*w*, *d*(CH_2_)], 1099 [*vs*, *n*(CO)]. ^1^H NMR (400 MHz in CD_2_Cl_2_): 3.62 (*s*, crown ether) ppm. ^13^C(^1^H) NMR (101 MHz in CD_2_Cl_2_): 122.4 (CN, ^1^ *J*_Pt—C_ = 1018 Hz), 70.1 (crown) ppm.

## Refinement   

Crystal data, data collection and structure refinement details are summarized in Table 2 ▸. The H atoms were placed geometrically with a constrained C—H distance of 0.99 Å and refined as riding atoms with *U*_iso_(H) = 1.2*U*_eq_(C).

## Supplementary Material

Crystal structure: contains datablock(s) I. DOI: 10.1107/S2056989019015238/hb4322sup1.cif

Structure factors: contains datablock(s) I. DOI: 10.1107/S2056989019015238/hb4322Isup2.hkl

CCDC references: 1965195, 1965195

Additional supporting information:  crystallographic information; 3D view; checkCIF report

## Figures and Tables

**Table 1:** Hydrogen-bond geometry (Å, °)

*D*—H⋯*A*	*D*—H	H⋯*A*	*D*⋯*A*	*D*—H⋯*A*
C3—H3*A*⋯N1^i^	0.99	2.54	3.510 (3)	165
C9—H9*B*⋯N2^ii^	0.99	2.55	3.459 (3)	152

Symmetry codes: (i) 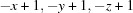; (ii) 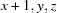.

**Table 2:** Experimental details

Crystal data	
Chemical formula	[K_2_Pt(CN)_4_(C_12_H_24_O_6_)_2_]
*M*_r_	905.99
Crystal system, space group	Monoclinic, *P*2_1_/*n*
Temperature (K)	100
*a*, *b*, *c* (Å)	11.7341 (10), 13.7280 (12), 11.8876 (10)
β (°)	94.999 (3)
*V* (Å^3^)	1907.6 (3)
*Z*	2
Radiation type	Mo *K*α
μ (mm^−1^)	3.96
Crystal size (mm)	0.44 × 0.44 × 0.12

Data collection	
Diffractometer	Bruker APEXII CCD
Absorption correction	Multi-scan (*SADABS*; Bruker, 2016 ▸)
*T*_min_, *T*_max_	0.306, 0.564
No. of measured, independent and observed [*I* > 2σ(*I*)] reflections	57788, 5839, 4658
*R*_int_	0.047
(sin θ/λ)_max_ (Å^−1^)	0.716

Refinement	
*R*[*F* ^2^ > 2σ(*F* ^2^)], *wR*(*F* ^2^), *S*	0.019, 0.051, 1.05
No. of reflections	5839
No. of parameters	215
H-atom treatment	H-atom parameters constrained
Δρ_max_, Δρ_min_ (e Å^−3^)	1.25, −1.54

Computer programs: *APEX2* and *SAINT* (Bruker, 2016 ▸), *SHELXS* (Sheldrick, 2008 ▸), *SHELXL2018* (Sheldrick, 2015 ▸), *OLEX2* (Dolomanov *et al.*, 2009 ▸) and *publCIF* (Westrip, 2010 ▸).

## supplementary crystallographic information

### Crystal data

**Table:**

[K_2_Pt(CN)_4_(C_12_H_24_O_6_)_2_]	*F*(000) = 912
*M**_r_* = 905.99	*D*_x_ = 1.577 Mg m^−^^3^
Monoclinic, *P*2_1_/*n*	Mo *K*α radiation, λ = 0.71073 Å
*a* = 11.7341 (10) Å	Cell parameters from 9630 reflections
*b* = 13.7280 (12) Å	θ = 2.3–30.6°
*c* = 11.8876 (10) Å	µ = 3.96 mm^−^^1^
β = 94.999 (3)°	*T* = 100 K
*V* = 1907.6 (3) Å^3^	Block, colourless
*Z* = 2	0.44 × 0.44 × 0.12 mm

### Data collection

**Table:**

Bruker APEXII CCD diffractometer	4658 reflections with *I* > 2σ(*I*)
φ and ω scans	*R*_int_ = 0.047
Absorption correction: multi-scan (SADABS; Bruker, 2016)	θ_max_ = 30.6°, θ_min_ = 2.3°
*T*_min_ = 0.306, *T*_max_ = 0.564	*h* = −16→16
57788 measured reflections	*k* = −19→19
5839 independent reflections	*l* = −17→16

### Refinement

**Table:**

Refinement on *F*^2^	Hydrogen site location: inferred from neighbouring sites
Least-squares matrix: full	H-atom parameters constrained
*R*[*F*^2^ > 2σ(*F*^2^)] = 0.019	*w* = 1/[σ^2^(*F*_o_^2^) + (0.0186*P*)^2^ + 1.9161*P*] where *P* = (*F*_o_^2^ + 2*F*_c_^2^)/3
*wR*(*F*^2^) = 0.051	(Δ/σ)_max_ = 0.001
*S* = 1.05	Δρ_max_ = 1.25 e Å^−^^3^
5839 reflections	Δρ_min_ = −1.54 e Å^−^^3^
215 parameters	Extinction correction: SHELXL2018 (Sheldrick, 2015), Fc^*^=kFc[1+0.001xFc^2^λ^3^/sin(2θ)]^-1/4^
0 restraints	Extinction coefficient: 0.0120 (4)
Primary atom site location: structure-invariant direct methods	

### Special details

**Table:**

Geometry. All esds (except the esd in the dihedral angle between two l.s. planes) are estimated using the full covariance matrix. The cell esds are taken into account individually in the estimation of esds in distances, angles and torsion angles; correlations between esds in cell parameters are only used when they are defined by crystal symmetry. An approximate (isotropic) treatment of cell esds is used for estimating esds involving l.s. planes.

### Fractional atomic coordinates and isotropic or equivalent isotropic displacement parameters (Å^2^)

**Table:**

	*x*	*y*	*z*	*U*_iso_*/*U*_eq_	
Pt1	0.500000	0.500000	0.000000	0.01307 (4)	
K1	0.81107 (3)	0.50085 (2)	0.38736 (3)	0.01684 (7)	
O5	0.93998 (11)	0.65091 (10)	0.29546 (12)	0.0220 (3)	
O2	0.71541 (11)	0.34705 (10)	0.50409 (12)	0.0232 (3)	
O4	0.97580 (12)	0.45517 (10)	0.23439 (12)	0.0256 (3)	
O3	0.82893 (12)	0.31294 (10)	0.30722 (12)	0.0254 (3)	
O1	0.68912 (13)	0.54392 (11)	0.57027 (12)	0.0258 (3)	
O6	0.84409 (13)	0.68057 (10)	0.50316 (12)	0.0273 (3)	
C2	0.34516 (17)	0.47354 (16)	0.05042 (17)	0.0234 (4)	
N2	0.25437 (16)	0.45768 (17)	0.07632 (17)	0.0358 (4)	
N1	0.61178 (17)	0.50228 (13)	0.25023 (17)	0.0284 (4)	
C9	0.99412 (18)	0.53486 (16)	0.15999 (18)	0.0268 (4)	
H9A	0.922679	0.549960	0.112661	0.032*	
H9B	1.054051	0.517845	0.109679	0.032*	
C1	0.56960 (16)	0.50133 (12)	0.15887 (17)	0.0192 (3)	
C5	0.68672 (17)	0.26882 (14)	0.42770 (18)	0.0265 (4)	
H5A	0.628172	0.290733	0.368065	0.032*	
H5B	0.654414	0.214014	0.468750	0.032*	
C10	1.03093 (17)	0.62118 (15)	0.23170 (19)	0.0276 (4)	
H10A	1.098625	0.603787	0.283356	0.033*	
H10B	1.052471	0.675460	0.182981	0.033*	
C4	0.61633 (18)	0.38299 (16)	0.5518 (2)	0.0309 (4)	
H4A	0.579757	0.329734	0.591811	0.037*	
H4B	0.560472	0.407414	0.491136	0.037*	
C6	0.79142 (17)	0.23559 (14)	0.37517 (17)	0.0250 (4)	
H6A	0.852386	0.218411	0.434705	0.030*	
H6B	0.773614	0.177181	0.328031	0.030*	
C8	0.9510 (2)	0.36800 (15)	0.1721 (2)	0.0335 (5)	
H8A	1.016517	0.350456	0.129004	0.040*	
H8B	0.882998	0.377767	0.117934	0.040*	
C14	0.7268 (2)	0.62271 (15)	0.64242 (18)	0.0308 (4)	
H14A	0.795873	0.603522	0.691114	0.037*	
H14B	0.666168	0.640928	0.691447	0.037*	
C11	0.97505 (19)	0.72802 (15)	0.37123 (18)	0.0295 (4)	
H11A	1.002946	0.783790	0.328515	0.035*	
H11B	1.038243	0.705558	0.425670	0.035*	
C12	0.8756 (2)	0.75891 (14)	0.43299 (19)	0.0311 (4)	
H12A	0.896408	0.816886	0.479879	0.037*	
H12B	0.810247	0.776359	0.378360	0.037*	
C7	0.9286 (2)	0.28768 (15)	0.2533 (2)	0.0329 (5)	
H7A	0.916538	0.225258	0.212284	0.039*	
H7B	0.994834	0.280173	0.310186	0.039*	
C3	0.65005 (18)	0.46399 (16)	0.63312 (18)	0.0280 (4)	
H3A	0.583590	0.484034	0.673536	0.034*	
H3B	0.711603	0.441769	0.689539	0.034*	
C13	0.7534 (2)	0.70708 (15)	0.5689 (2)	0.0342 (5)	
H13A	0.684720	0.724656	0.518624	0.041*	
H13B	0.775999	0.764336	0.616299	0.041*	

### Atomic displacement parameters (Å^2^)

**Table:**

	*U*^11^	*U*^22^	*U*^33^	*U*^12^	*U*^13^	*U*^23^
Pt1	0.01451 (5)	0.01192 (5)	0.01299 (6)	0.00149 (3)	0.00243 (3)	−0.00065 (3)
K1	0.01690 (16)	0.01710 (16)	0.01701 (17)	−0.00121 (12)	0.00425 (13)	−0.00143 (13)
O5	0.0216 (6)	0.0192 (6)	0.0254 (7)	−0.0033 (5)	0.0037 (5)	−0.0028 (5)
O2	0.0223 (6)	0.0220 (6)	0.0260 (7)	−0.0027 (5)	0.0065 (5)	−0.0029 (5)
O4	0.0296 (7)	0.0201 (7)	0.0283 (7)	0.0010 (5)	0.0104 (6)	−0.0033 (6)
O3	0.0289 (7)	0.0185 (6)	0.0301 (8)	0.0011 (5)	0.0105 (6)	−0.0019 (5)
O1	0.0319 (7)	0.0256 (7)	0.0213 (7)	−0.0017 (6)	0.0103 (6)	−0.0027 (6)
O6	0.0382 (8)	0.0194 (6)	0.0255 (7)	−0.0025 (6)	0.0099 (6)	−0.0032 (5)
C2	0.0251 (9)	0.0275 (9)	0.0176 (8)	0.0004 (7)	0.0018 (7)	−0.0031 (7)
N2	0.0260 (9)	0.0547 (13)	0.0273 (9)	−0.0040 (9)	0.0062 (7)	−0.0055 (9)
N1	0.0228 (8)	0.0430 (11)	0.0193 (8)	0.0027 (7)	0.0013 (7)	−0.0011 (7)
C9	0.0278 (10)	0.0273 (9)	0.0272 (10)	0.0016 (8)	0.0138 (8)	0.0007 (8)
C1	0.0156 (8)	0.0228 (9)	0.0193 (8)	0.0009 (6)	0.0030 (6)	−0.0009 (6)
C5	0.0282 (10)	0.0226 (9)	0.0291 (10)	−0.0070 (7)	0.0052 (8)	−0.0030 (8)
C10	0.0202 (9)	0.0268 (9)	0.0373 (11)	−0.0026 (7)	0.0101 (8)	0.0013 (8)
C4	0.0261 (10)	0.0301 (10)	0.0390 (12)	−0.0041 (8)	0.0167 (9)	−0.0026 (9)
C6	0.0300 (10)	0.0180 (8)	0.0268 (10)	−0.0017 (7)	0.0020 (8)	−0.0013 (7)
C8	0.0426 (12)	0.0252 (10)	0.0355 (12)	−0.0025 (9)	0.0194 (10)	−0.0103 (9)
C14	0.0422 (12)	0.0276 (10)	0.0243 (10)	0.0007 (9)	0.0131 (9)	−0.0070 (8)
C11	0.0360 (11)	0.0226 (9)	0.0301 (10)	−0.0121 (8)	0.0037 (8)	−0.0037 (8)
C12	0.0475 (13)	0.0174 (9)	0.0292 (10)	−0.0060 (8)	0.0075 (9)	−0.0046 (8)
C7	0.0376 (11)	0.0207 (9)	0.0427 (13)	0.0024 (8)	0.0169 (10)	−0.0080 (9)
C3	0.0280 (10)	0.0310 (10)	0.0273 (10)	0.0011 (8)	0.0162 (8)	0.0006 (8)
C13	0.0496 (13)	0.0227 (10)	0.0322 (11)	0.0032 (9)	0.0147 (10)	−0.0070 (8)

### Geometric parameters (Å, º)

**Table:**

Pt1—C2^i^	1.996 (2)	C9—C10	1.501 (3)
Pt1—C2	1.996 (2)	C5—H5A	0.9900
Pt1—C1^i^	1.991 (2)	C5—H5B	0.9900
Pt1—C1	1.991 (2)	C5—C6	1.497 (3)
K1—K1^ii^	4.9761 (9)	C10—H10A	0.9900
K1—O5	2.8308 (14)	C10—H10B	0.9900
K1—O2	2.8133 (14)	C4—H4A	0.9900
K1—O4	2.8369 (14)	C4—H4B	0.9900
K1—O3	2.7642 (14)	C4—C3	1.503 (3)
K1—O1	2.7691 (14)	C6—H6A	0.9900
K1—O6	2.8354 (14)	C6—H6B	0.9900
K1—N1	2.732 (2)	C8—H8A	0.9900
K1—C4	3.527 (2)	C8—H8B	0.9900
O5—C10	1.422 (2)	C8—C7	1.503 (3)
O5—C11	1.427 (2)	C14—H14A	0.9900
O2—C5	1.428 (2)	C14—H14B	0.9900
O2—C4	1.425 (2)	C14—C13	1.500 (3)
O4—C9	1.435 (3)	C11—H11A	0.9900
O4—C8	1.424 (2)	C11—H11B	0.9900
O3—C6	1.427 (2)	C11—C12	1.493 (3)
O3—C7	1.425 (2)	C12—H12A	0.9900
O1—C14	1.426 (2)	C12—H12B	0.9900
O1—C3	1.426 (3)	C7—H7A	0.9900
O6—C12	1.429 (2)	C7—H7B	0.9900
O6—C13	1.421 (3)	C3—H3A	0.9900
C2—N2	1.155 (3)	C3—H3B	0.9900
N1—C1	1.154 (3)	C13—H13A	0.9900
C9—H9A	0.9900	C13—H13B	0.9900
C9—H9B	0.9900		
			
C2^i^—Pt1—C2	180.0	O2—C5—H5B	109.7
C1—Pt1—C2	91.47 (8)	O2—C5—C6	109.75 (16)
C1^i^—Pt1—C2^i^	91.47 (8)	H5A—C5—H5B	108.2
C1—Pt1—C2^i^	88.53 (8)	C6—C5—H5A	109.7
C1^i^—Pt1—C2	88.53 (8)	C6—C5—H5B	109.7
C1^i^—Pt1—C1	180.0	O5—C10—C9	109.71 (16)
O5—K1—K1^ii^	74.34 (3)	O5—C10—H10A	109.7
O5—K1—O4	59.78 (4)	O5—C10—H10B	109.7
O5—K1—O6	59.94 (4)	C9—C10—H10A	109.7
O5—K1—C4	160.31 (5)	C9—C10—H10B	109.7
O2—K1—K1^ii^	96.09 (3)	H10A—C10—H10B	108.2
O2—K1—O5	170.43 (4)	K1—C4—H4A	159.0
O2—K1—O4	118.28 (4)	K1—C4—H4B	82.0
O2—K1—O6	117.20 (4)	O2—C4—K1	49.29 (9)
O2—K1—C4	22.59 (4)	O2—C4—H4A	109.8
O4—K1—K1^ii^	73.68 (3)	O2—C4—H4B	109.8
O4—K1—C4	139.85 (5)	O2—C4—C3	109.49 (17)
O3—K1—K1^ii^	95.19 (3)	H4A—C4—H4B	108.2
O3—K1—O5	119.15 (4)	C3—C4—K1	82.54 (11)
O3—K1—O2	61.00 (4)	C3—C4—H4A	109.8
O3—K1—O4	59.77 (4)	C3—C4—H4B	109.8
O3—K1—O1	121.99 (4)	O3—C6—C5	108.32 (16)
O3—K1—O6	165.50 (5)	O3—C6—H6A	110.0
O3—K1—C4	80.51 (5)	O3—C6—H6B	110.0
O1—K1—K1^ii^	94.33 (3)	C5—C6—H6A	110.0
O1—K1—O5	118.53 (4)	C5—C6—H6B	110.0
O1—K1—O2	61.13 (4)	H6A—C6—H6B	108.4
O1—K1—O4	167.99 (5)	O4—C8—H8A	109.9
O1—K1—O6	59.40 (4)	O4—C8—H8B	109.9
O1—K1—C4	42.15 (5)	O4—C8—C7	108.77 (18)
O6—K1—K1^ii^	70.44 (3)	H8A—C8—H8B	108.3
O6—K1—O4	115.57 (4)	C7—C8—H8A	109.9
O6—K1—C4	101.40 (5)	C7—C8—H8B	109.9
N1—K1—K1^ii^	175.95 (5)	O1—C14—H14A	110.2
N1—K1—O5	102.88 (5)	O1—C14—H14B	110.2
N1—K1—O2	86.68 (5)	O1—C14—C13	107.71 (17)
N1—K1—O4	102.40 (5)	H14A—C14—H14B	108.5
N1—K1—O3	83.55 (5)	C13—C14—H14A	110.2
N1—K1—O1	89.59 (5)	C13—C14—H14B	110.2
N1—K1—O6	110.92 (5)	O5—C11—H11A	109.9
N1—K1—C4	76.81 (6)	O5—C11—H11B	109.9
C4—K1—K1^ii^	106.82 (4)	O5—C11—C12	109.03 (17)
C10—O5—K1	116.61 (11)	H11A—C11—H11B	108.3
C10—O5—C11	111.15 (15)	C12—C11—H11A	109.9
C11—O5—K1	115.51 (11)	C12—C11—H11B	109.9
C5—O2—K1	109.45 (11)	O6—C12—C11	109.03 (17)
C4—O2—K1	108.12 (11)	O6—C12—H12A	109.9
C4—O2—C5	110.99 (15)	O6—C12—H12B	109.9
C9—O4—K1	111.94 (11)	C11—C12—H12A	109.9
C8—O4—K1	113.54 (11)	C11—C12—H12B	109.9
C8—O4—C9	110.80 (17)	H12A—C12—H12B	108.3
C6—O3—K1	117.58 (11)	O3—C7—C8	107.85 (17)
C7—O3—K1	118.16 (11)	O3—C7—H7A	110.1
C7—O3—C6	112.30 (15)	O3—C7—H7B	110.1
C14—O1—K1	118.68 (12)	C8—C7—H7A	110.1
C3—O1—K1	117.32 (11)	C8—C7—H7B	110.1
C3—O1—C14	111.44 (16)	H7A—C7—H7B	108.4
C12—O6—K1	113.75 (11)	O1—C3—C4	108.13 (17)
C13—O6—K1	114.36 (12)	O1—C3—H3A	110.1
C13—O6—C12	111.85 (16)	O1—C3—H3B	110.1
N2—C2—Pt1	177.97 (18)	C4—C3—H3A	110.1
C1—N1—K1	146.76 (17)	C4—C3—H3B	110.1
O4—C9—H9A	110.2	H3A—C3—H3B	108.4
O4—C9—H9B	110.2	O6—C13—C14	109.05 (17)
O4—C9—C10	107.64 (17)	O6—C13—H13A	109.9
H9A—C9—H9B	108.5	O6—C13—H13B	109.9
C10—C9—H9A	110.2	C14—C13—H13A	109.9
C10—C9—H9B	110.2	C14—C13—H13B	109.9
N1—C1—Pt1	178.81 (18)	H13A—C13—H13B	108.3
O2—C5—H5A	109.7		

Symmetry codes: (i) −*x*+1, −*y*+1, −*z*; (ii) −*x*+2, −*y*+1, −*z*+1.

### Hydrogen-bond geometry (Å, º)

**Table:**

*D*—H···*A*	*D*—H	H···*A*	*D*···*A*	*D*—H···*A*
C3—H3*A*···N1^iii^	0.99	2.54	3.510 (3)	165
C9—H9*B*···N2^iv^	0.99	2.55	3.459 (3)	152

Symmetry codes: (iii) −*x*+1, −*y*+1, −*z*+1; (iv) *x*+1, *y*, *z*.
